# Cell-to-cell proteome variability: life in a cycle

**DOI:** 10.1038/s41392-021-00655-8

**Published:** 2021-06-10

**Authors:** Tsz Kin Suen, Burcu Al, Katarzyna Placek

**Affiliations:** Immunology and Metabolism, Life and Medical Sciences Institute, University of Bonn, Bonn, Germany

**Keywords:** Cell biology, Cancer, Gene expression analysis

Increasing body of evidence, supported by single-cell transcriptomic analysis, points to the heterogeneity of single cells within same cell population. In the recent study by Mahdessian et al.^1^ published in *Nature* the authors elegantly show the cell-to-cell variability in the protein expression, its (in)dependence on the cell cycle progression and provide with the list of new cycling proteins with a potential role in tumorigenesis.

An immense development of next-generation sequencing techniques in the recent decade enabled the characterization of the whole transcriptome at a single-cell level. The single-cell RNA-sequencing (scRNA-seq) analysis has revealed a heterogeneity of cells within one cell population at the mRNA level.^2^ This elicits the question of whether the cell heterogeneity also occurs at the protein level. The question which is challenging to answer due to the technical limitations of a single-cell proteomic analysis. Most of the techniques used to assess expression of specific proteins at a single-cell level, such as imaging, flow cytometry, or mass cytometry (CyTOF), rely on the affinity reagent: specific antibodies. Mass spectrometry offers the measurement of protein abundance without the use of antibodies, and therefore is an uprising promise of unbiased single-cell proteomics. Encouraged by the first successful attempts of implementing mass spectrometry to characterize single mammalian cells (SCoPE-MS) this technology now awaits further developments.^3^

**Fig. 1:**
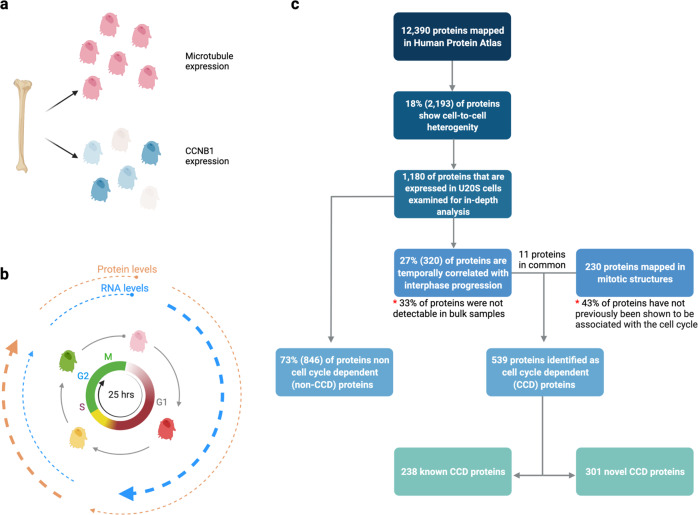
Cell cycle regulates expression profile of certain proteins. **a** Cell-to-cell variability in protein expression in U2OS cells exemplified by the specific cell-cycle-dependent (CCD) cyclin B1 (CCNB1), in blue, and homogenously expressed microtubules, in pink. **b** Fluorescent ubiquitination-based cell cycle indicator (FUCCI) system in U2OS cells: each cell temporarily expresses two fluorescently tagged cell cycle markers (i) Chromatin Licensing and DNA Replication Factor 1 (CDT1) expressed in G1 phase and tagged with red fluorescent protein (RFP) (ii) Gemini (GMNN) expressed during S and G2 phases and tagged with green fluorescent protein (GFP). Both markers expressed in S-transition so yellow fluorescent is detected. Most of CCD transcripts peak in expression during G1 phase, blue circle, while most CCD proteins peak during S and G2 phases, orange circle. **c** Flow chart summarizes the numbers and percentages of the proteins examined in the study. As a first step, proteins that show cell-to-cell variability in terms of protein expression are selected from an open-access comprehensive resource of mapped 12,390 proteins, Human Protein Atlas. 18% of all proteins show cell-to-cell variability. Two hundred and thirty of these proteins localize to mitotic structures in U2OS cells. Thousand one hundred and eighty proteins displaying cell-to-cell variability were further characterized using the FUCCI system, so a group of 320 proteins whose expression levels are correlated with cell cycle progression is identified. The 230 proteins whose expression correlates with the cell cycle progression are summed up with the ones expressed in mitotic structures and are called cell cycle dependent (CCD) proteins. Out of 539 CCD proteins, 238 have been studied and 301 have not been associated with cell cycle progression, they were named “known CCD” and “novel CCD” proteins, respectively. Eight hundred and forty-six proteins that are not correlated with the cell cycle are non-CCD proteins. Text labeled with asterisks indicates the state of the art resulting from studies using the FUCCI system and imaging techniques. Created with Biorender.com

Human Protein Atlas (HPA) serves as a data source of levels and subcellular localization of thousands of proteins in various human cell types. This huge collection of immunofluorescence and confocal microscopy images revealed that one-fifth of the proteome displays cell-to-cell heterogeneity in expression level or spatial distribution in a variety of human cell lines (Fig. 1a). Based on this observation, Mahdessian et al. investigated whether this proteome variability shows a temporally controlled expression pattern in correlation to cell cycle progression.^1^ The authors applied targeted single-cell imagining analysis using fluorescent ubiquitination-based cell cycle indicator (FUCCI) in human bone U2OS cells. FUCCI is a refined cell cycle monitoring system that relies on the cycling expression pattern of two proteins: gemini, which is expressed in the S, G2, and M phases of the cell cycle and was fused to the green fluorescent protein (GFP); and chromatin licensing and DNA replication factor 1 (CDT1), which is expressed in G1 phase and was tagged with the red fluorescent protein (RFP). The dynamic fluorescence change of the nuclei from red in phase G1, through yellow in S phase, to green in phases G2 and M allows the segregation of cells based on the cell cycle phases^4^ (Fig. 1b). Using the aforementioned method, Mahdessian et al. created a spatiotemporally resolved map of proteome in unsynchronized cycling human cells and provided the list of cell cycle dependent (CCD) proteins, most of them have not been described so far.^1^ In-depth analysis of 1180 proteins in U2OS cells revealed that less than a half of the proteins with cell-to-cell variability display a correlation with the cell cycle progression, while the majority of proteome with cell-to-cell variability cannot be explained by cell cycling (non-CCD) (Fig. 1c). The authors further suggested that non-CCD proteomic variation may be biologically relevant to other cellular processes, such as metabolism.

In order to further investigate whether the CCD proteins were temporally regulated by transcript cycling, the scRNA-seq was employed and gene expression was determined according to pseudotimes calculated with the FUCCI model. While some proteins (e.g., Anillin Actin Binding Protein, ANLN) showed a similar temporal profile of abundance to the temporal profile of their RNA expression during cell cycle progression, most of the proteins (e.g., Scinderin, SCIN) did not show such correlation, indicating that other mechanisms such as translational or post-translational modifications (PTMs) may largely regulate the temporal dynamics of proteomic cycling (Fig. 1b). The observed dissociation of the protein abundance cycling from the transcript abundance is in accordance with previous reports.^5^ It is estimated from a bulk analysis that 40% of the variability in protein levels can be explained by the variability in transcript level. This emphasizes the need of proper profiling of gene expression at both: mRNA and protein levels when investigating molecular mechanisms.

The differential regulation of CCD and non-CCD proteins by PTMs was further investigated by comparing the kinases upstream of phosphosites on these proteins. The kinase family which regulates cell fate was overrepresented upstream of phosphosites on CCD proteins, whereas kinase family which regulates diverse pathways including metabolism was overrepresented upstream of phosphosites on non-CCD proteins.^1^ The former observation was true for CCD proteins both with and without cycling transcripts indicating that PTMs may assist the regulation of CCD proteins cycling regardless of transcripts cycling, whereas the latter observation suggests that the PTM regulation links non-CCD protein variations to other pathways such as metabolism. While the authors focused on the regulation of protein abundance by phosphorylation, other PTMs, such as ubiquitination, that lead to protein degradation and therefore regulate protein levels, could be also involved in the process. Moreover, the impact of non-coding transcripts on protein levels could be worth-investigating.

Finally, Mahdessian et al. addressed the functional importance of the novel CCD proteins for cellular proliferation and their potential as therapeutic targets for cancer treatment.^1^ The bulk mRNA sequencing across human healthy tissues and cancers showed that the novel CCD proteins were expressed at higher levels in proliferative tissues than non-proliferative ones. Furthermore, the authors found that the CCD proteins correspond to genes that are enriched in factors necessary for the cell survival, and confirmed this hypothesis by implementing short interfering RNA (siRNA)-mediated gene silencing on selected novel CCDs. As cellular proliferation is important for cancerogenesis, the study revealed an overrepresented association of CCD protein production with the survival of cancer patients. The immunohistochemical staining of few CCD proteins in healthy and cancer tissues confirmed the potential gene candidates involved in tumorigenesis, such as Family With Sequence Similarity 50 Member B (FAM50B) gene or CD2 Cytoplasmic Tail Binding Protein 2 (CD2BP2). All this evidence confirms that the novel CCD proteins may have a functional role in cell proliferation, and might be a potential target for cancer therapy.

In summary, by applying a proteogenomic analysis Mahdessian et al. have generated a cellular atlas of hundreds of novel CCD proteins and their corresponding transcripts. Their work provides great insights into the functional understanding of cell-to-cell variability, and an inspiration for new single-cell multiomics measurements. The potential application of these novel CCD proteins may act as new clinical markers for cancer diagnosis.
